# Nutritional Status in Patients with Medication-Related Osteonecrosis of the Jaw (MRONJ)

**DOI:** 10.3390/nu13051585

**Published:** 2021-05-10

**Authors:** Johannes Laimer, Alexander Höller, Ute Pichler, Raphael Engel, Sabrina B. Neururer, Alexander Egger, Andrea Griesmacher, Emanuel Bruckmoser

**Affiliations:** 1 University Hospital for Craniomaxillofacial and Oral Surgery, Medical University Innsbruck, A-6020 Innsbruck, Austria; johannes.laimer@i-med.ac.at (J.L.); raphael.engel@student.i-med.ac.at (R.E.); 2 Service for Nutrition and Dietetics, University Hospital Innsbruck, Medical University Innsbruck, A-6020 Innsbruck, Austria; alexander.hoeller@tirol-kliniken.at (A.H.); ute.pichler@tirol-kliniken.at (U.P.); 3 Department of Clinical Epidemiology, Tyrolean Federal Institute for Integrated Care, Tirol Kliniken GmbH, A-6020 Innsbruck, Austria; s.neururer@tirol-kliniken.at; 4 Central Institute for Medical and Chemical Laboratory Diagnosis, University Hospital Innsbruck, Medical University Innsbruck, A-6020 Innsbruck, Austria; alexander.egger@tirol-kliniken.at (A.E.); andrea.griesmacher@i-med.ac.at (A.G.); 5 Private Practice for Oral and Maxillofacial Surgery, A-5020 Salzburg, Austria

**Keywords:** medication-related osteonecrosis of the jaw, MRONJ, nutritional status, malnutrition, micronutrients, macronutrients, nutritional therapy, wound healing

## Abstract

Medication-related osteonecrosis of the jaw (MRONJ) is a potentially severe side effect of mostly antiresorptive drugs. The aim of this prospective clinical study was to evaluate the nutritional status in MRONJ patients scheduled for surgical treatment (intraoral soft tissue closure). The following parameters were evaluated: body weight, body height, BMI, nutritional risk index (NRI), bioelectric impedance analysis (BIA), vitamins A, B12, D3, E, K1, folic acid, iron, total protein, transferrin, ferritin, prealbumin, albumin, and zinc. All subjects were admitted to hospital four to five days before surgery and sip-fed with Nutritia Fortimel Compact Protein in addition to regular oral food intake. During surgery, a nasogastric tube was inserted and only removed on hospital discharge five days postoperatively. A total of 58 patients could be included. Half of the MRONJ patients were identified to be at risk for malnutrition. Deficiencies regarding protein levels were revealed, whereas hardly any relevant deficits of micronutrients were noted. The intraoral wound healing four weeks post-surgery was highly satisfactory with a low dehiscence rate of intraoral mucosal sites. Of all parameters analyzed, the dehiscence rate at the last follow-up four weeks post-surgery was significantly influenced by vitamin K, transferrin, and ferritin levels (*p* = 0.030, *p* = 0.004, and *p* = 0.023, respectively). In conclusion, perioperative dietary counselling and appropriate nutritional therapy are important supportive measures in MRONJ patients scheduled for intraoral soft tissue closure.

## 1. Introduction

### 1.1. Medication-Related Osteonecrosis of the Jaw (MRONJ)

Medication-related osteonecrosis of the jaw (MRONJ) is a potential side effect of antiresorptive agents which are mainly used for osteoporosis treatment or in tumor patients with osseous metastases. Exposed areas of necrotic jaw bone, pain, infection, and a high recurrence rate (with dehiscence of intraoral mucosa) after surgical soft tissue closure represent the main clinical features and challenges of this condition [1]. The risk of developing MRONJ is considerably higher in oncologic compared to osteoporotic patients with up to 2.8 percent for oncologic [2], and up to 0.03 percent for osteoporotic patients, respectively [3].

MRONJ was first seen as a side effect in patients receiving bisphosphonates and was initially referred to as BRONJ (bisphosphonate-related osteonecrosis of the jaw) [4,5]. Bisphosphonates are widely used since the 1990s to treat osteoporosis, hypercalcemia of malignancy, Paget disease of the bone, and malignancies with osseous metastases [6]. The same pattern of osteonecrosis was later found in patients receiving the monoclonal antibody denosumab [7] which is used for prevention of skeletal-related events (bone pain, fractures, etc.) secondary to multiple myeloma or bone metastases from solid tumors, giant cell tumor, hypercalcemia in malignancies, and osteoporosis [8]. In recent years, anti-angiogenetic drugs and tyrosine kinase inhibitors have also been reported to cause jaw osteonecrosis as a drug side effect [9]. Consequently, this condition is now referred to as medication-related osteonecrosis of the jaw (MRONJ) to account for the different types of causative drugs [10] including tyrosine kinase inhibitors, monoclonal antibodies, mammalian target of rapamycin inhibitors, radiopharmaceuticals, selective estrogen receptor modulators, and immunosuppressants [11]. Recently, a possible correlation between Tocilizumab (a humanized antiinterleukin-6-receptor monoclonal antibody) and MRONJ has been reported [12].

MRONJ therapy remains controversial and no international consensus on the best treatment strategy has been established yet. One of the main challenges is the high postoperative dehiscence rate following intraoral soft tissue closure. Purely conservative approaches are hardly successful and mostly aim at alleviation of symptoms only. Apart from classical conservative or surgical management, adjuvant therapies for MRONJ including leukocyte- and platelet-rich fibrine, laser ablation, and fluorescence-guided surgery might have potential in improving the healing process [13]. Regarding platelet concentrates, current evidence is not sufficient to establish the effectiveness of these autologous blood derivates in the prevention and treatment of MRONJ [14], although therapeutic benefits have been reported in subjects suffering from facial cutaneous sinus tracts secondary to MRONJ [15].

Considering that subjects suffering from MRONJ are predominantly tumor patients in palliative care, a twofold problem is typical in this collective. On the one hand, many patients show a reduced nutritional status following a protracted course of their metastatic tumor disease. On the other hand, adequate food intake is often difficult if MRONJ complicates the underlying oncologic disease.

As indicated above, MRONJ can also occur in osteoporosis patients, although the frequency and risk is significantly lower compared to tumor patients. Since nutrition is closely related to bone, muscle and joint health, it is not surprising that osteoporosis patients may be at risk of malnutrition [16].

### 1.2. Malnutrition

The concept of malnutrition differentiates between disease-associated malnutrition with and without the presence of inflammatory processes as well as between acute and chronic malnutrition. In oncologic patients, malnutrition presents as inflammatory, chronic disease [17]. The typical status is characterized by insufficient nutritional supply and, at the same time, the need for an increased supply due to hypermetabolism, loss of nutrients, and malabsorption. Deficits can be of both quantitative (e.g., energy, proteins, carbohydrates, lipids) and qualitative (e.g., vitamins, minerals, micronutrients) in nature.

Due to differing definitions, the prevalence of malnutrition reported in the international literature ranges between 20 and 60 percent [18,19]. Consequences of malnutrition include both higher morbidity and mortality [20] which can be deduced from health-economic figures, such as increased length of hospital stays [18,21] as well as higher complication and re-admission rates [22,23]. Early detection of malnutrition is key for effective and efficient nutritional therapy.

### 1.3. Malnutrition and Cancer

According to the National Cancer Institute (NCI), up to 80% of cancer patients are affected by malnutrition, which is responsible for nearly 20 percent of cancer-related deaths [24]. Due to an inadequate dietary intake, oncologic patients may suffer from various complications. These can include longer hospital stays and impaired quality of life as well as lower survival rates. The reasons for malnutrition are manifold comprising metabolic changes and insufficient food intake due to mucositis, dysphagia, vomiting, and diarrhea. A total of 80% of patients with advanced cancer suffer from cachexia accounting for almost one in three cancer-related deaths.

### 1.4. Malnutrition and Osteoporosis

Amongst elderly people, osteoporosis represents a healthcare issue of great importance. During aging, a gradual loss of bone mass leads to osteopenia and osteoporosis. Although both sexes are concerned, menopausal women are affected to a much larger extent. The risk of skeletal fractures and further morbidity and mortality are considerably higher in subjects suffering from osteoporosis [25]. Risk factors for this bone-affecting disease include decreased physical activity, a lack of sex hormones, as well as genetic and environmental influences.

Malnutrition is a common finding in elderly people, and nutritional deficiencies play an important role for the development of osteoporosis [26]. Calcium supplementation can reduce both the loss of bone density and the frequency of fractures in older people [27]. As another potential risk factor, reduced protein intake leading to lower bone density is discussed [28]. In addition to calcium and its co-player vitamin D, the importance of vitamin K for skeletal integrity is uncontested. Surprisingly, the international literature provides only a few publications specifically dealing with the nutritional status in osteoporotic patients.

### 1.5. Nutrition and Wound Healing

Wound healing is a complex process of cellular and biochemical events that considerably depend on the nutritional substrates available. The wound healing phase is very demanding with regard to energy requirements. The significant increase in enzyme activity, protein synthesis, and cell proliferation during the healing process requires both building substrates and energy. Under usual circumstances, these substrates are released from protein reserves and body energy stores. In undernourished patients, increased food intake or supplements with high energy and protein density are required. The protein metabolism plays a key role here, and the protein demand can significantly increase depending on a patient’s health condition. In addition to basic macronutrients (amino acids, proteins, carbohydrates, fat), electrolytes and micronutrients are essential.

Due to the lack of endogenous substrates, wound healing is delayed during periods of starvation (simple or stress starvation). If the nutritional status is not taken into account, the patient’s ability to heal may be compromised. Consequently, the stages of wound healing may be prolonged. Further undernutrition negatively impacts wound healing in addition to a prolonged phase of inflammation, delayed neovascularization, decreased collagen synthesis, dysfunction of B and T cells, decreased phagocytosis (leukocytes), and decreased mechanical strength of the skin [29].

In order to provide additional energy for wound healing, it is key that adequate amounts of nutrients are provided. Fatty acids are not only required for cell structures (such as membranes) but also play an important role in the inflammatory process. Wound healing depends on good nutrition including polyunsaturated fatty acids. Protein deficiency is known to contribute to poor wound healing with reduced collagen formation and higher rates of wound dehiscence. Significant exudate loss can lead to protein deficiency. Vitamins also play an important role in wound healing [30,31]. Even short periods of malnutrition can negatively affect proper wound healing. Therefore, nutritional deficiencies must be recognized in a timely manner so that repletion can be initiated as soon as possible.

### 1.6. Micronutrients

Micronutrients play a key role in various metabolic processes that promote survival from critical illness. Vitamins are, for example, involved in oxidative phosphorylation, which is altered in case of systemic inflammation. Vitamins can provide protection against mediators, such as oxidants. Apart from vitamins, there are trace elements essential for direct antioxidant activity. They also serve as cofactors for various antioxidant enzymes. Adequate levels of both vitamins and trace elements are key for wound healing and immune function.

### 1.7. Proteins

Proteins play an essential role during the whole wound healing process [29]. Cells of the immune system including lymphocytes, monocytes, and macrophages mainly consist of proteins. These cells are required for the initiation of a healthy inflammatory response in the healing process. Therefore, it is obvious that sufficient protein supply is essential for proper wound healing. A lack of proteins leads to decreased collagen synthesis, and collagen is the main protein in the healing wound.

### 1.8. Prealbumin

Prealbumin (or transthyretin) serves as transport protein for thyroid hormones [32]. A serum prealbumin concentration of < 10 mg/dL can indicate a state of malnutrition. Prealbumin levels can also be reduced in case of infection, liver dysfunction, over-hydration, and physiological stress. Prealbumin is especially useful as a marker in the elderly and during refeeding. It has several advantages over albumin. Due to its shorter half-life of only two to three days, it can better reflect acute changes with regard to the nutritional status. Furthermore, it is not influenced by intestinal protein loss in cases of protein-losing enteropathies. Elevated levels of prealbumin may be found in cases of renal dysfunction, corticosteroid therapy or dehydration.

### 1.9. Albumin

The serum albumin level is known as a nutritional marker and a marker for healing processes [33]. It has been evaluated as an indicator of wound healing in several observational studies [34,35]. In these studies, low serum albumin levels were associated with slower wound healing and/or poor prognosis of the healing process. In one study, low serum albumin levels (below 35 g/L) were significantly associated with poor prognosis of leg ulcer healing [34]. In another study, low serum albumin levels (< 4 g%) were associated with impaired skin graft uptake [36]. Yet another study revealed that wound healing was delayed in open fractures of the lower limb in patients with low serum albumin levels [35].

### 1.10. Anthropometric Measurements

Anthropometrics comprise techniques and methods to measure and evaluate specific features, such as body height and weight including data interpretation and categorization of the respective results. Body measurements play a key role in the assessment of a person’s nutritional status.

One of the principal aims when evaluating anthropometric measurements in nutrition medicine is the quick and simple assessment of the nutritional status. From primary measurement data, further parameters can be derived. It needs to be emphasized that evaluated and calculated parameters must always be interpreted in the whole individual context since no single parameter can be regarded in an isolated way for assessing the nutritional status.

#### 1.10.1. Body Mass Index (BMI)

BMI (body mass index) is a commonly used indicator for the assessment of the weight-related nutritional status and the body fat mass. It is derived from the weight in kilograms and the height in meters by dividing the body mass through the square of the body height (kg/m^2^). The BMI is the most frequently used indicator to assess body mass. It allows for categorization into underweight, normal weight, and overweight. In this context, normal weight is defined as the range (in kg/m^2^) with the lowest mortality rate and the highest life expectancy. Overall, it lies between 18.5 and 25 kg/m^2^. However, the respective range shifts towards higher readings (24 to 29) with increasing age.

#### 1.10.2. Bioelectrical Impedance Analysis (BIA) and Bioelectrical Impedance Vector Analysis (BIVA)

BIA (bioelectrical impedance analysis) is a simple and non-invasive technique for the evaluation of body composition. Body compartments include total body water, lean body mass, fat mass, fat free mass, muscle mass, body cell mass, and extracellular mass. BIA is based on the measurement of electric resistance when an alternating current is connected to the body via specific electrodes. This procedure allows to determine the impedance parameters resistance (R) and reactance (Xc), i.e., capacitative resistance. As a compound parameter, the phase angle can be directly derived from R and Xc. It provides information about tissue hydration, cell membrane integrity, and intercellular connections. Taking into account additional parameters such as sex, age, weight, and height, it is possible to determine the body composition using specific formulas that are based on the assumption that the hydration status of the fat free mass is 73% [37].

BIVA (bioelectrical impedance vector analysis) is a more advanced approach when analyzing raw data of BIA measurements. The results can be graphically illustrated with a bivariate vector, which is calculated from resistance (R) and reactance (Xc) normalized per body height (H), i.e., R/H and Xc/H, respectively. This allows for a weight-independent interpretation of results. The length and direction of this vector can indicate hydration status (edema or dehydration) and body cell mass, thereby allowing for assessment of the body composition even in case of an altered hydration status. Using BIVA, a reliable differentiation between obese and athletic as well as between cachectic and lean individuals is possible.

### 1.11. Malnutrition Screening Using Questionnaires

The Nutritional Risk Screening 2002 (NRS-2002) score was conceived by the Danish Association of Parenteral and Enteral Nutrition (DAPEN) and is recommended to assess the risk for malnutrition, especially in an inpatient setting. Following a pre-screening with four questions (weight, weight loss, food intake, underlying disease), the necessity for a detailed evaluation (main screening) is determined.

A detailed nutritional risk assessment includes the following parameters: (I) severity of impact of the primary disease on the nutritional status, (II) weight change within the last one to three months, (III) changes in food intake within one week, (IV) body mass index (BMI), and (V) age > 70 years. Each of these parameters scores 1 when positive. A widely accepted threshold for an elevated risk of malnutrition is an NRS score ≥ 3 [38].

### 1.12. Study Aim

The aim of this study was to assess the preoperative nutritional status in MRONJ patients scheduled for intraoral soft tissue closure, and to describe its course during postoperative follow-up. Furthermore, a potential correlation between a reduced nutritional status and impaired wound healing was to be evaluated. To the best of our knowledge, this is the first study assessing the nutritional status of this highly vulnerable patient group in recent literature.

## 2. Materials and Methods

In this prospective single-center study, MRONJ patients scheduled for intraoral soft tissue closure at the University Hospital for Craniomaxillofacial and Oral Surgery in Innsbruck, Austria were invited to participate. Subjects suffering from MRONJ stages 1, 2, or 3 according to the AAOMFS (American Association of Oral and Maxillofacial Surgeons) classification [1] were eligible for inclusion. The enrolment period started in October 2018 and ended in August 2020.

Exclusion criteria included a severely impaired general health condition where patients would not be able to tolerate the dietetic assessment. Due to possible interference with impedance measurements, patients with ICDs (implantable cardioverter-defibrillator) were also excluded.

Written informed consent was obtained from each patient who agreed to participate in this study following a detailed explanation of the procedural workflow. Prior to any patient enrolment, the study had been approved by the institutional board in charge, i.e., the ethics committee of the Medical University Innsbruck, Austria. The respective reference number was 1190/2018. The study was conducted in full accordance with the principles expressed in the declaration of Helsinki.

For primary assessment of the current nutritional status, the NRS-2002 was used. Various parameters including body weight, body height, BMI, impaired general health status, unintended weight loss, reduced food intake, and current status of the underlying disease were evaluated. From this data, the respective score was calculated. NRS-2002 readings ≥3 were considered as risk factor for malnutrition requiring dietetic intervention. Additionally, the nutritional risk index (NRI) was calculated: NRI = (1.519 × serum albumin, g/dL) + {41.7 × present weight (kg)/ideal body weight (kg)}. According to their results, patients were separated into four categories [39]: major risk (NRI < 83.5), moderate risk (NRI 83.5–97.5), mild risk (NRI 97.5–100), and no risk (NRI > 100).

For qualitative assessment of potential malnutrition—and irrespective of the NRS-2002 screening results—a bioelectric impedance analysis (BIA) using the BIACORPUS RX 4000 (MEDI CAL HealthCare GmbH, Karlsruhe, Germany) was performed for all subjects. Physiological reference values for body cell mass range from 27 to 39.2 kg for men, and from 15 to 20.9 kg for women. The normal target range for phase angle readings lies between 5.5° and 7.0° for men, and between 5.0° and 6.3° for women. The physiological range for body fat mass ranges from 8.9 to 12.9 kg in women, and from 10.4 to 19.1 kg in men. Normal values for the cellular part as indicated in the international literature lie between 50 and 56 percent. Reference values for extracellular water (ECW) range from 38 to 44 percent in men, and from 39 to 45 percent in women.

In addition, blood samples were taken to determine the following parameters potentially influencing wound healing: vitamin D3 (in serum), vitamins A and E (both in EDTA plasma) as well as vitamin K1 (in EDTA plasma) were determined by high-performance liquid chromatography (HPLC) using commercial kits from Chromsystems Instruments & Chemicals GmbH (Graefelfing, Germany) and Immundiagnostik AG (Bensheim, Germany), respectively. A cobas 8000 analyzer (Roche Diagnostics, Rotkreuz, Switzerland) was used to determine vitamin B12 and folic acid levels immunologically in serum. Iron and total protein were photometrically analyzed in Li-heparin plasma. Transferrin, ferritin, and albumin were turbidimetrically assessed in Li-heparin plasma. Zinc was measured in Li-heparin plasma using reagents from Labor + Technik (Eberhard Lehmann GmbH, Berlin, Germany). Prealbumin was determined nephelometrically on a BNII analyzer (Siemens Healthcare Diagnostics GmbH, Munich, Germany) using reagents from Siemens. All analyses were performed at hospital admission (t1), hospital discharge (t2), and about one month after surgery (t4) when patients returned for the final follow-up visit. No blood samples were taken at t3 when sutures were removed about 14 days postoperatively. All blood samples were taken in the morning after overnight fasting.

Routine preoperative management for MRONJ patients was as follows: all subjects were admitted to hospital four to five days before surgery to start with IV antibiotics. In general, a combination of ampicillin and sulbactam (Pfizer, New York City, NY, USA) was administered. In case of a known allergy to penicillin, clindamycin (Pfizer, New York City, NY, USA) was used. Patients were sip-fed with two drinks of Nutritia Fortimel Compact Protein (18 g, 300 kcal) in addition to regular oral food intake. During the surgical intervention including removal of necrotic jawbone followed by intraoral soft tissue closure, a nasogastric tube was inserted and only removed on hospital discharge five days postoperatively. During this time, IV antibiotics were continued, and patients were fed with Novasource GI Control (Nestle, Vevey, Vaud, Switzerland) through the nasogastric tube. Apart from being sip-fed with two doses of 300 to 400 kcal/18 to 20 g protein, they were only allowed to drink water and tea. The standardized regimen from surgery to hospital discharge was as follows: 275 kcal with 10.25 g protein on day 0 (surgery), 550 kcal with 20.5 g protein on the first postoperative day (day 1), 825 kcal with 30.8 g protein on day 2, 1100 kcal with 41.0 g protein on day 3, and 1650 kcal with 61.5 g protein on day 4. On day 5, patients were discharged from hospital and were prescribed oral antibiotics for one month until the final follow-up (t4).

### Statistics

For a basic description of the patient collective, a descriptive statistical analysis is provided. Metrically scaled variables are indicated as mean ± standard deviation (SD), and median with interquartile ranges. Qualitative variables are given as absolute and relative frequencies. Data were explored for normal distribution using the Kolmogorov–Smirnov test. Intra-group differences were analyzed using the *t*-test for paired samples and Wilcoxon’s rank sum test. For qualitative group comparisons, the McNemar test was used. The Bonferroni method was used to correct for multiple testing. Therefore, *p*-values < 0.017 were statistically significant for multiple comparisons as indicated. For all statistical analyses, SPSS 26 (IBM Corp., Armonk, NY, USA) was used.

## 3. Results

### 3.1. Patient and Disease Specific Characteristics

In total, 61 subjects were primarily eligible for inclusion in this study. Of these, two patients who could not be operated on due to high-grade aortic stenosis had to be excluded. One patient has withdrawn their consent to study participation and had to be excluded as well. One patient died before the scheduled evaluation at t4; thus, only assessments at t1 and t2 could be included in the statistical analysis.

Basic characteristics of the study population are depicted in Table 1.

48 MRONJ patients (83%) were suffering from multiple myeloma or tumors with osseous metastases. Nine subjects (15%) with osteoporosis and one patient (2%) undergoing corticoid treatment due to osteoporosis prophylaxis could also be included in this study (Table 2).

According to the AAOMFS classification as updated in 2014 [1], 13 patients (22%) were classified as stage I, 42 patients (72%) as stage II, and 3 patients (5%) as stage III (Table 3).

A total of 32 (67%) tumor patients received 120 mg of Denosumab every four weeks. In 12 (25%) tumor patients, antiresorptive therapy was switched from intravenous bisphosphonates to 120 mg of Denosumab during the course of their malignancy. Three (6%) patients received intravenous bisphosphonates every four weeks. Osteoporotic patients (nine subjects) were given oral bisphosphonates in 33%, IV bisphosphonates in 44%, and Denosumab 60 mg twice a year in 22% of cases. An overview is provided in Table 4.

Postoperative wound dehiscence was not seen in any patient at t2 but was diagnosed in eight patients (14%) at t3, and in 10 subjects (17%) at t4 (Table 5).

### 3.2. Nutritional Risk Screening 2002 (NRS-2002)

According to the NRS-2002 threshold of ≥3 identifying patients at risk with need for individually tailored nutritional therapy, a risk for malnutrition was revealed in 33 MRONJ patients (57%). Details are provided in Table 6.

### 3.3. Nutritional Risk Index (NRI)

The NRI was available in 57 subjects. Seventeen patients (29%) were at risk at t1, 27 of 57 subjects (47%) were at risk at t2, and 11 of 49 individuals (22%) at t4.

### 3.4. Bioelectrical Impedance Analysis (BIA)

BIA measurement data were available in 57 patients at t1, in 54 subjects at t2, and in 49 individuals at t4. Readings at t1 were pathologically low in two patients (4%) and too high in 42 subjects (74%). Thirteen individuals (23%) showed physiologically normal readings. At t2, four patients (7%) were below, and 39 subjects (72%) were above physiological reference levels. Eleven individuals (20%) showed normal readings at t2. Readings at t4 were too low in one patient (2%) and too high in 38 subjects (78%). Ten individuals (20%) showed normal readings at t4.

Regarding the body cell mass (BCM), readings were below normal in 14 patients (26%) at t1, in 13 subjects (24%) at t2, and in 10 individuals (20%) at t4. Readings in the remainder were within the physiological range. At t1, an average phase angle of 4.6° was seen. This reading significantly increased to 4.9° post-surgery (*p* = 0.001); however, it did not reach normal values. At t4, average readings of 4.5° were found. Details are provided in Table 7.

### 3.5. Laboratory Results

#### 3.5.1. Total Protein

A total of 14 MRONJ patients (26%) had subnormal levels of total protein at t1, whereas 40 subjects (74%) showed normal readings. The mean serum level was 7.1 ± 0.7 g/dL. Up to the first postoperative measurement (t2), a significant deterioration of total protein levels was seen (*p* = 0.001) with a mean of 6.9 ± 0.7 g/dL. At this point, 25 patients (46%) showed subnormal values, whereas 29 subjects (54%) had normal readings. Following discharge from hospital, readings improved with mean serum protein levels of 7.1 g/dL corresponding to the initial mean concentration at hospital admission. Ten individuals (19%) still showed serum protein levels below the normal range. A total of 43 participants (81%) had serum protein levels within the normal range at t4, which was significantly higher (*p* < 0.001) as compared to the corresponding levels at t2.

#### 3.5.2. Prealbumin

At t1, prealbumin levels were normal in 45 patients (78%) and below the physiological range in 13 subjects (22%). The mean value was 22.8 ± 5.8 mg/dL. At t2, the mean prealbumin level significantly decreased (*p* = 0.006) to 21.0 mg/dL. At this point, 33 patients (57%) had normal values, whereas readings were below normal in 25 subjects (43%). Compared to t2, increased prealbumin levels were found at t4 with a mean of 25.6 ± 6.7 mg/dL. At t4, 43 patients (80%) had normal levels, whereas 11 subjects (20%) showed prealbumin levels below normal. Compared to the initial value at t1, readings at t2 and t4 were significantly higher (*p* = 0.001, and *p* = 0.004, respectively).

#### 3.5.3. Albumin

At t1, the mean serum albumin level was 3742 ± 465 mg/dL with 39 patients (67%) being in the normal range, and 19 subjects (33%) showing values below normal. During the stay in hospital, the mean serum albumin level significantly decreased to 3493 ± 487 mg/dL at t2 (*p* = 0.001). At this point, 24 patients (41%) had a normal serum albumin level, whereas 34 subjects (59%) showed readings below normal. At t4, the mean serum albumin level had increased to 3865 ± 407 mg/dL, corresponding to a significant improvement as compared to t1 (*p* = 0.006) and t2 (*p* = 0.001).

#### 3.5.4. Vitamins

Regarding the serum levels of vitamins A, D3, E, K1, B12, and folic acid, only a few patients showed deficiencies at t1: two patients (4%) for vitamin B12, eight subjects (15%) for folic acid, 35 participants (60%) for vitamin D3, and none for vitamins A or K1. The levels of vitamin B12 and folic acid significantly increased during the course of the nutritional intervention (*p* = 0.001 for both vitamins). For all other vitamins, serum levels remained stable showing no significant difference to t2 or t4 as compared to the starting point at t1.

Despite considerable food restrictions (soft diet with mashed food), vitamin A, D3, E, K1 levels at t4 were found to be within normal range. Except for vitamin K1, which showed a significant improvement from 813 ± 496 ng/L at t1 to 1228 ± 880 ng/L at t4, no significant difference was found for the other vitamins when comparing t4 serum levels to readings at t1 or t2. Vitamin D3 levels were found to be slightly below normal throughout all assessments which probably corresponds to average values in the healthy population. Table 5 shows serum level changes of vitamins including levels of significance.

#### 3.5.5. Zinc and Iron Metabolism

Mean values for zinc, iron, transferrin and ferritin were within normal range throughout (i.e., at t1, t2, and t4). Iron and transferrin levels significantly decreased from t1 to t2 (*p* = 0.005, and *p* = 0.001, respectively) and recovered at t4 (*p* = 0.005, and *p* = 0.002). Ferritin levels significantly increased from t1 to t2 (*p* = 0.004), whereas the respective readings significantly decreased from t2 to t4 (*p* < 0.001) and from t1 to t4 (*p* = 0.017). For zinc levels, no statistically significant changes were found.

#### 3.5.6. Nutritional Factors Influencing Wound Healing

Of all parameters analyzed, vitamin K, transferrin, and ferritin significantly influenced the dehiscence rate at t4 (*p* = 0.030, *p* = 0.004, and *p* = 0.023, respectively). Details are provided in Table 8.

## 4. Discussion

In this prospective non-controlled clinical study, we describe for the first time the nutritional status in MRONJ patients undergoing surgery for intraoral soft tissue closure. Furthermore, the potential influence of a preoperatively compromised nutritional status on the outcome of the surgical treatment has been evaluated. To assess the course and development of possible nutritional deficiencies, and to evaluate potential benefits of nutritional therapy, the nutritional status was determined before surgery (t1), directly after surgery (t2), and four weeks postoperatively (t4). In this pilot study, 58 subjects were included for both nutritional screening and in-depth nutritional assessment comprising numerous laboratory analyses as well as bioelectrical impedance measurements.

Most MRONJ patients suffer from multiple myeloma or tumors with osseous metastases as an underlying disease. Much less frequently, this side effect of mainly antiresorptive drugs is seen in osteoporotic patients. Since both tumor and osteoporotic subjects frequently show nutritional deficiencies [40], these patients should be considered as a highly vulnerable collective. Tumor patients often show nutritional deficiencies resulting in poor overall survival, impaired quality of life, longer hospital stays, higher costs for the healthcare system, and a greater likelihood of hospital readmission [41,42].

To date, there is no gold standard to assess disease-associated nutritional deficiencies [43]. The international literature describes various screening tools, laboratory analyses, and physical tools such as bioelectrical impedance analysis (BIA). One of the most popular and most widely used screening tools is the NRS-2002 [44], which was also used in our study. A total of 56% of all subjects showed an elevated risk for malnutrition (NRS ≥ 3). This finding in our patient collective is in accordance with other studies investigating tumor patients (including but not limited to head and neck tumors) [45,46]. In hospitalized patients in general, the rate of nutritional deficiencies ranges between 20 and 60 percent.

Regarding electrophysiological measurements, BIA represents a non-invasive and cost-effective technique to evaluate body composition including respective changes over time. This method has already been studied in tumor patients for long-term observations [47,48]. On the one hand, assessments at a single point in time are reported [49]. On the other hand, the effect of nutritional therapy on the nutritional status over time is described [47,50]. Furthermore, scientific data suggest a correlation of certain BIA parameters (especially the phase angle) with the overall survival rate [47].

During the five weeks of each patient’s study period, the focus was clearly set on the perioperative investigations and nutritional status assessment. A great part of the patient collective showed an elevated fat mass throughout the study period. A quarter of the subjects had a low BCM preoperatively. The results in our patients demonstrate that body weight and fat mass alone are not suitable for assessing the nutritional status. Evaluating the body compartments separately using BIA measurements allows for a further differentiation of the nutritional status.

One of the best indicators for cellular health is the phase angle, where higher values reflect better cell function and a stronger cell membrane. There is ongoing discussion on whether the phase angle is a suitable parameter to describe changes in the nutritional status [51]. Cut-off values of < 6° are seen as an indicator for malnutrition. In our collective, average phase angle readings at the beginning of the study period were relatively low (4.6°). Under nutritional therapy, this value significantly increased postoperatively. However, with average readings of 4.9°, this result is still below the normal range. Three weeks after surgery, a decrease of the respective values was noted with an average reading of 4.5°. The low phase angle readings in our patients presumably point to a generally compromised health status in this collective.

In the international literature, deficiencies regarding various macro- and micronutrients in disease-associated malnutrition are sometimes controversially discussed. These deficiencies are reflected in pathological readings of laboratory analyses and can potentially affect proper wound healing. To assess the utility of these parameters in the context of MRONJ management, total protein, prealbumin, albumin, iron, ferritin, transferrin, zinc, folic acid, and vitamins A, D, D3, E, K1, and B12 were analyzed during the course of our study.

In the past, serum total protein and some specific serum proteins (e.g., prealbumin, albumin) have been widely used to determine the nutritional status. However, since many disease processes can alter prealbumin and albumin levels, these parameters alone do not represent reliable serum markers for malnutrition [52,53,54].

In our study, 14 patients (26%) had a total serum protein below normal, in 19 subjects (33%) albumin levels were too low, and 13 individuals (22%) showed prealbumin levels below the physiological range at the beginning of the study period (t1). In 14 patients (26%), transferrin levels were too low at t1. The mean values ±SD at the beginning of our study were as follows: total protein 7.1 ± 0.7 g/dL, albumin 3742 ± 465 mg/dL, prealbumin 22.8 ± 5.9 mg/dL, and transferrin 230 ± 45 mg/dL.

Directly after surgery (t2), considerably more patients showed serum levels below normal: 25 patients (45%) for total protein, 34 subjects (59%) for albumin, 25 individuals (43%) for prealbumin, and 18 participants (31%) for transferrin. These changes were found to be statistically significant for total protein (*p* = 0.001), albumin (*p* < 0.001), prealbumin (*p* = 0.006), and transferrin (*p* = 0.001).

At the follow-up four weeks post-surgery (t4), only 10 patients (19%) had a total protein below normal, 11 subjects (20%) showed prealbumin and albumin levels below the normal range, and in six individuals (11%) transferrin was found to be too low. Plausible reasons for the postoperative decrease of the respective serum levels could be the surgical trauma as well as the higher demand during wound healing (27174435). Proteins undoubtedly play a key role in the growth and repair of wounds. Proteins are constantly deposited into new cells to replace lost protein. New living tissue requires protein; thus, no wound can heal without adequate protein supply [55].

Except for vitamin D3, only a few subjects in our study collective showed a lack of micronutrients, such as vitamins A, E, K1, B12, folic acid, zinc, or iron at baseline. Directly after surgery, a significant decrease was seen for iron (*p* = 0.005). However, this decrease below normal reference values only concerned a few patients (see Table 5). A potentially elevated need for iron due to bleeding during surgery could be a reasonable explanation for this finding.

The NRI represents a combination of laboratory parameters and anthropometric assessments. At the first evaluation, this index had a mean value of 104.7. Two patients (3%) were considered as being at major risk, 12 subjects (21%) at moderate risk, three individuals (5%) at mild risk, and 41 participants (71%) were not at risk with regard to their NRI readings. Following standardized nutritional therapy, deterioration at t2 was noted and attributed to increased protein consumption from the healing wounds. Rapid recovery at t4 under continued ONS (oral nutritional supplement) therapy in an outpatient setting underlines the importance of ONS and could indicate the benefit of nutritional supplements early before hospital admission. This approach could compensate for preexisting protein deficits.

Regarding the BMI—which is an integral part of the NRI—a significant postoperative decrease (*p* < 0.001) could be noted, followed by a significant recovery (*p* = 0.011) four weeks post-surgery.

Our pilot study showed a significant correlation at t4 between wound healing (dehiscence rate) and ferritin, transferrin, and vitamin K. This result must be interpreted with care due to the relatively limited number of study participants and the low wound dehiscence rate (17%) at t4. For clinical practice, the focus should not solely be on singular laboratory parameters but should take into account the whole picture of the nutritional status.

## 5. Conclusions

This prospective clinical study is the first of its kind to show that more than half of the MRONJ patients scheduled for surgical treatment are at risk for malnutrition and should be offered nutritional counselling.

As discussed abundantly in the international literature, the principal challenge is to establish a suitable method to assess the nutritional status and diagnose malnutrition. The NRS-2002 as an easy-to-perform assessment seems to well reflect the clinical picture and is highly suitable in daily routine from a practical point of view. Therefore, we advocate the application of the NRS-2002 in all MRONJ patients at hospital admission or even before. Since deficiencies regarding protein levels were striking in our patient collective, total protein, prealbumin, albumin, and transferrin should be closely monitored, and the amino acid profile to be substituted should be individually adapted to the respective patients’ requirements. It appears advisable to begin with these measures in an outpatient setting already in order to establish a physiological protein status well before hospital admission. Providing a non-individualized nutritional therapy for about five days postoperatively using a nasogastric tube resulted in a stable nutritional status of the assessed parameters with the exception of protein levels.

Since hardly any relevant deficits of micronutrients were noted, we do not consider a detailed assessment of these parameters essential for routine clinical practice. Additionally, BIA does not provide relevant additional information for adaption of the dietary regime. Under general nutritional therapy, the wound healing after four weeks post-surgery is highly satisfactory, reflected by a relatively low dehiscence rate. Since more than half of the patients were shown to be at risk for malnutrition even before hospital admission, surgery for MRONJ entailing postoperative restrictions for oral food intake can even further complicate nutritional management. Therefore, perioperative dietary counselling and appropriate nutritional therapy are essential in MRONJ patients scheduled for intraoral soft tissue closure. Further studies are needed to verify the correlation between nutritional status and outcomes of MRONJ surgical treatment.

## Figures and Tables

**Table 1:** Basic characteristics of the study population (*n* = 58).

Variables	Readings
Age (years) mean (range)	70.5 (42; 86)
Female	31 (53%)
Male	27 (47%)
Weight (cm) ± SD	71.5 ± 15.4
Height (kg) ± SD	168.8 ± 8.9
BMI ± SD	25.1 ± 4.8
Smoking (yes)	8 (14%)
Alcohol (yes)	20 (35%)

BMI: body mass index, SD: standard deviation.

**Table 2:** Underlying diseases/indications for antiresorptive therapy (*n* = 58).

Diseases	Absolute Numbers	Percentage
Breast cancer	18	31%
Prostate cancer	13	22%
Lung cancer	5	9%
Multiple myeloma	11	19%
Colon carcinoma	1	2%
Osteoporosis	10	17%

**Table 3:** MRONJ stages in absolute numbers and percent.

MRONJ Stage	Absolute Numbers	Percentage
MRONJ stage 1	13	22%
MRONJ stage 2	42	72%
MRONJ stage 3	3	5%

MRONJ: medication-related osteonecrosis of the jaw.

**Table 4:** Type of antiresorptive medication (*n* = 58).

Drugs	Absolute Numbers	Percentage
Denosumab	27	47%
Bisphosphonate	10	17%
Denosumab and Bisphosphonate	21	36%

**Table 5:** Intraoral mucosal status at different points in time (absolute numbers and percentages).

Time Points	Closed Wound		Dehiscence	
t2	58	100%	0	0%
t3	50	86%	8	14%
t4	47	83%	10	17%

t2: hospital discharge, t3: removal of sutures, t4: one month after surgery. At t4, one patient’s status was missing.

**Table 6:** Absolute numbers and percentages of NRS scores (*n* = 58).

NRS Score	Absolute Numbers	Percentage
0	4	7%
1	7	12%
2	14	24%
3	18	31
4	10	17%
5	4	7%
6	1	2%

NRS: nutritional risk screening.

**Table 7:** Anthropometric measurements at different points in time (t1, t2, t4). *p*-values < 0.017 are statistically significant.

	t1 MRONJ		t2 MRONJ		*p*-Value (t1 MRONJ vs. t2 MRONJ)	t4 MRONJ		*p*-Value (t2 MRONJ vs. t4 MRONJ)	*p*-Value (t1 MRONJ vs. t4 MRONJ)
	Mean	SD	Mean	SD		Mean	SD		
**Phase angle**	4.6	1.1	4.9	3.6	**0.001**	4.5	1.0	0.847	**0.007**
**BIA fat mass**	31.6	8.5	31.3	8.7	0.823	31.1	8.0	0.788	0.155
**BIA fat mass (kg)**	23.2	9.4	22.5	9.3	**0.004**	22.3	8.5	0.380	0.019
**BIA BCM (kg)**	21.3	6.5	20.1	5.6	**0.000**	21.0	6.3	**0.009**	0.139
**BIA cell mass**	43.6	6.5	42.5	6.3	**0.003**	43.4	6.5	0.073	0.264
**BIA ECWI**	19.0	4.4	18.3	3.6	0.171	18.5	3.5	0.075	0.389
**BIA ECW (%)**	53.0	6.3	54.1	6.1	**0.002**	53.1	6.2	0.040	0.330
**FFM (kg)**	48.2	9.6	47.0	8.7	**0.000**	47.6	9.3	**0.015**	0.032
**BMI**	25.1	4.8	24.4	4.6	**0.000**	24.6	4.5	**0.011**	**0.003**
**NRI**	104.7	11.6	99.6	11.6	**0.000**	106.2	10.2	**0.000**	0.384

MRONJ: medication-related osteonecrosis of the jaw; SD: standard deviation; BIA: bioelectrical impedance analysis; BCM: body cell mass; ECW: extracellular water; ECWI: extracellular water in liters; FFM: fat free mass (in kilograms); BMI: body mass index; NRI: nutritional risk index; significant *p*-values in bold.

**Table 8:** Blood serum values of vitamins, proteins, and micronutrients at different points in time (t1, t2, t4). *p*-values < 0.017 are statistically significant.

	t1 MRONJ		t2 MRONJ		*p*-Value (t1 MRONJ vs. t2 MRONJ)	t4 MRONJ		*p*-Value (t2 MRONJ vs. t4 MRONJ)	*p*-Value (t1 MRONJ vs. t4 MRONJ)
	Mean	SD	Mean	SD		Mean	SD		
**Vitamin B12**	329.7	167.6	477.5	272.7	**0.000**	391.5	261.2	**0.000**	**0.001**
**Folic acid**	8.3	5.3	10.0	4.6	**0.000**	8.3	3.9	**0.001**	0.096
**Vitamin K**	813.3	496.7	1021.7	728.5	0.074	1227.5	879.8	0.105	**0.000**
**Vitamin A**	0.5	0.2	0.5	0.2	0.127	0.6	0.2	**0.002**	0.018
**Vitamin E**	15.6	4.0	15.6	3.2	0.951	17.1	4.6	0.037	0.068
**Vitamin D3**	70.4	40.1	71.3	36.7	0.626	74.1	33.7	0.291	0.095
**Zinc**	11.3	1.6	15.4	27.7	0.281	11.3	1.6	0.564	0.583
**Iron**	14.4	7.0	12.0	4.8	**0.005**	14.5	6.4	**0.005**	0.825
**Transferrin**	230.2	45.3	216.9	44.9	**0.001**	244.3	46.3	**0.000**	**0.002**
**Ferritin**	298.2	438.0	301.9	482.1	**0.004**	209.0	244.9	**0.000**	0.017
**Prealbumin**	22.8	5.9	21.0	5.3	**0.006**	25.6	6.7	**0.000**	**0.004**
**Albumin**	3742.1	465.4	3492.5	486.5	**0.000**	3865.4	407.4	**0.000**	**0.006**
**Total protein**	7.1	0.7	6.8	0.7	**0.001**	7.1	0.6	**0.000**	0.849

MRONJ: medication-related osteonecrosis of the jaw; SD: standard deviation; significant *p*-values in bold.

## Data Availability

Raw data will be made available upon reasonable request.

## Abbreviations

AAOMFS	American Association of Oral and Maxillofacial Surgeons
BCM	Body Cell Mass
BIA	Bioelectric Impedance Analysis
BIVA	Bioelectrical Impedance Vector Analysis
BMI	Body Mass Index
DAPEN	Danish Association of Parenteral and Enteral Nutrition
ECW	Extracellular Water
ECWI	Extracellular Water in Liters
EDTA	Ethylenediaminetetraacetic Acid
FFM	Fat Free Mass
H	Body Height
HPLC	High-performance Liquid Chromatography
ICD	Implantable Cardioverter-defibrillator
MRONJ	Medication-related Osteonecrosis of the Jaw
NCI	National Cancer Institute
NRI	Nutritional Risk Index
NRS	Nutritional Risk Screening
ONS	Oral Nutritional Supplement
R	Resistance
SD	Standard Deviation
Xc	Reactance
